# Exploring the potential of the carbon credit program for hedging energy prices in Brazil

**DOI:** 10.1007/s11356-024-32387-x

**Published:** 2024-02-17

**Authors:** Rafael Baptista Palazzi, Derick David Quintino, Paulo Jorge Silveira Ferreira, Festus Victor Bekun

**Affiliations:** 1 Getulio Vargas Foundation (FGV), São Paulo School of Business Administration, Sao Paulo, Brazil; 2 Independent Researcher, Nova Odessa, SP 13380-009 Brazil; 3 VALORIZA—Research Center for Endogenous Resource Valorization, 7300-555 Portalegre, Portugal; 4 Department of Economic Sciences and Organizations, Polytechnic Institute of Portalegre, 7300-555 Portalegre, Portugal; 5 Center for Advanced Studies in Management and Economics, Palácio Do Vimioso, Largo Marquês de Marialva, 8, 7000-809 Évora, Portugal; 6 Faculty of Economics Administrative and Social Sciences, Istanbul Gelisim University, Istanbul, Türkiye; 7 Adnan Kassar School of Business, Department of Economics, Lebanese American University, Beirut, Lebanon; 8 Development Economis Research Center, Azerbaijan State University of Economics(UNEC), Istiqlaliyyat Str.6, 1001 Baku, Azerbaijan; 9 VALORIZA - Research Centre for Endogenous Resource Valorization, Polytechnic Institute of Portalegre, Madrid, Spain

**Keywords:** CBIO, Carbon reduction, Commodity, Commodity markets, Green energy, Hedge ratio, RenovaBio

## Abstract

The transition to a low-carbon economy is imperative to reduce reliance on fossil fuels and mitigate pollution emissions. This preposition also aligns with the United Nations Sustainable Development Goals (SDGs-13), which highlight the climate change action. In this vein, Brazil has implemented the Decarbonization Credit (CBIOS) program to incentivize biofuel production and promote environmental sustainability through carbon credit emissions. To this end, the present study evaluates the effectiveness of the CBIO contract as a hedging tool for investors in the face of energy price fluctuations and decarbonization efforts. Specifically, we employ conditional dynamic correlation (DCC-GARCH) and optimal hedge ratio (HR) techniques to assess the relationship between CBIO and the futures and spot prices of sugar, oil, and ethanol. Our findings suggest that the current CBIO contract is not an effective hedge against energy spot and future prices. However, our analysis identifies a strengthening correlation between ethanol traded in Chicago and CBIO over time, highlighting the potential for an underlying contract to serve as an effective hedging tool in the future. Our study adds to the existing literature on carbon pricing mechanisms and their impact on financial markets, emphasizing the importance of sustainable energy policies and their potential to mitigate the risks associated with energy price volatility and decarbonization efforts.

## Introduction

Most economies are presently grappling with an unprecedented energy crisis exacerbated by the ongoing conflict in Ukraine. During periods of heightened economic uncertainty, bond flows display heterogeneous behavior, with emerging countries attracting more capital than advanced economies due to higher yields (Balcilar et al. 2021). In this case, it may be a good opportunity for emerging countries to attract capital to finance green projects.

Despite the multitude of efforts implemented by developed economies, such as the Paris Agreement, aiming to mitigate their reliance on fossil fuels, countries continue to rely on such energy sources. This has policy implications not only for the supply of energy as populations grow but also renders it more susceptible to disruptions in the world economy. For instance, the findings of Olasehinde-Williams et al. (2023) suggest that transitioning from non-renewable to renewable energy use is one channel through which countries can address energy inflation triggered by geopolitical risks.

Moreover, some recent studies show the impact of oil prices on output growth (Alao et al. 2023), CO_2_ emissions (Ike et al. 2020a), and the role of the substitution effect of renewable energy prices from increasing non-renewable energy prices (Ike et al. 2020b; Shan et al. 2021). Thus, the reliance on fossil fuels raises significant concerns regarding greenhouse gas (GHG) emissions and air pollution. In response, countries have implemented mechanisms to attribute a value to carbon emissions, either through taxes or emission trading systems, aiming to facilitate the transition to a low-carbon economy. The European Union Emissions Trading System (EU ETS) stands as a pioneering example, and subsequently, carbon prices have evolved into part of the set of production factors, essentially becoming commodities (Demiralay et al. 2022).

Brazil has a distinctive position in the world regarding green fuel consumption. Brazilian consumers can choose between gasoline or ethanol when refueling their vehicles at gas stations throughout the country. Policies promoting ethanol consumption are essential to reduce dependence on imported fossil fuels and contribute to the environment due to its positive externalities, particularly the mitigation of greenhouse gas (GHG) emissions. Notably, using ethanol instead of gasoline can reduce GHG emissions by up to 90% (Macedo and Seabra 2008; de Sousa and Rodrigues 2020).

Implementation of the RenovaBio (Brazil’s National Biofuels Policy) in December, 2017, in Brazil aimed to reduce GHG emissions in accordance with the Paris Agreement. Besides the reduction of GHG emissions, aiming to achieve the targets established in the context of COP21, that program has a second primary goal: to promote the security of fuel supply in Brazil by incentivizing biofuel production (de Sousa and Rodrigues 2020).

This new legislation introduced a carbon pricing market known as the Decarbonization Credit (CBIOS), which operates through a carbon sequestration certificate obtained during the production of ethanol based on sugarcane. The CBIOS corresponds to the reduction of 1 ton of equivalent carbon dioxide compared to fossil fuel emissions. Ethanol producers employ a mathematical tool (RenovaCalc) to calculate this amount based on the product’s life cycle analysis. Fuel distributors, in turn, acquire these certificates, which can be resold to companies that need to meet environmental compensation targets (Chandel et al. 2021).

RenovaBio has achieved significant growth in credit commercialization. From 2020 to 2021, the volume of credits traded increased from R$ 14.89 million to R$ 29.8 million, resulting in a total of R$ 1.17 billion on the B3 Brazilian stock exchange (Mendes 2022). The price of CBIOS has reached a record high of R$ 104.50, which is 93.4% above its historical average of R$ 52.12 (NovaCana 2022a), showing that economic agents are optimistic about the future of decarbonization credit pricing.

The Brazilian exchange market, B3, plans to launch CBIOS future contracts in 2023. This development is expected to enhance market transparency and attract more market participants, ultimately strengthening liquidity in the future market. The introduction of CBIOS future contracts may also provide additional opportunities for investors to hedge their risks and manage their portfolios. The launch of these futures contracts is eagerly anticipated by market participants and is expected to play a vital role in developing and maturing the decarbonization credit market (Agência Estado 2022).

The transition toward a low-carbon economy necessitates incentives such as decarbonization credits, with CBIOS playing a crucial role in promoting cleaner energy production. However, there is a need for further research on the relationship between CBIOS prices and other critical variables in the energy market. The previous empirical literature has focused on studying Brazilian ethanol price relationships in light of institutional changes, such as Petrobras’ pricing policy, which aligns domestic prices with international reference prices. Several studies, including those by David et al. (2020), Hallack et al. (2020), Nascimento Filho et al. (2021), Quintino et al. (2022), and Palazzi et al. (2022), have investigated the impact of this policy on fuel prices. Additionally, Dutta and Bouri (2019) and Quintino et al. (2021a) have contributed to the understanding of price relationships of carbon emission prices in the context of the Brazilian sugarcane and ethanol sector.

Nevertheless, one of the crucial pillars sustaining the viability of CBIO relies on its adoption in the market. Insufficient liquidity poses a significant risk, potentially undermining the long-term effectiveness of this mechanism designed to reduce fossil fuel usage. Such a scenario could run counter to the goals of the carbon credit program. Therefore, the CBIO program could benefit from the alignment with other contracts. This strategy strengthens its resilience and enhances its attractiveness as a versatile contract capable of serving both as a hedging tool and an alternative investment (Demiralay et al. 2022).

The significance of hedging energy prices lies in how companies and countries navigate the considerable fluctuations in fuel prices. Not only are energy prices highly volatile but also their nonlinear behavior—such as responses to policy shifts, asymmetric reactions to news, and susceptibility to financial turbulence—underscores the importance of hedging as a mechanism to ensure price stability (Palazzi et al. 2024).

Moreover, Demiralay et al. (2022) highlight the advantages of using carbon future contracts for hedging. In addition to asset-level and country-level diversification as tools for risk mitigation, a diverse array of financial market participants, including institutional investors and other traders with an interest in the carbon market, view this market as a valuable investment opportunity. Their participation enhances liquidity in the market. Therefore, understanding the effectiveness of hedging can empower participants in these markets, enabling them to enhance risk management strategies and optimize portfolio risk.

Therefore, this study contributes to the literature on the CBIOS market by addressing a research gap and enhancing our understanding of how CBIOS contracts can be used to manage energy price risks. The relationship between CBIOS prices and cash prices for sugar and ethanol, Brent spot price, and future contracts is analyzed by employing the dynamic conditional correlation (DCC-GARCH) methodology to analyze the volatility dynamics of CBIOS paired with the primary underlying contracts. For example, Alao et al. (2023) underscore the advantage of using the DCC-GARCH methodology to model oil price volatility.

The optimal hedge ratio (HR) is also applied to evaluate the hedge performance of CBIOS against futures and cash prices. To build the out-of-sample one-step ahead forecasts, a rolling window is used. To the best of our knowledge, this study is the first attempt to measure the effectiveness of CBIOS as a hedging instrument in the Brazilian energy market, specifically in relation to primary commodity prices (both cash and futures).

It is worth noticing that a few studies have delved into dynamic hedging methodologies in the carbon market. For instance, Fan et al. (2013) assessed hedging performance in the European carbon market, and Demiralay et al. (2022) conducted an analysis of dynamic conditional correlation (DCC) to compute the dynamic hedge ratio, utilizing the IHS market global carbon index.

Our findings indicate that, at present, the CBIOS contract is not an effective hedge against energy spot and future prices. However, our optimal hedge analysis suggests a strengthening correlation between ethanol Platts and CBIOS over time, which could lead to the development of a useful underlying contract for hedging in the future. These results have significant implications for investors and policymakers seeking to manage the risks associated with energy prices and decarbonization efforts, as well as for the future viability of the CBIO contract.

The present work is structured as follows. Following this introductory section, the second section presents the methods and data employed, the third section discusses the results obtained, and finally, the fourth section presents final considerations.

## Method and data

### Dynamic conditional correlation (DCC) modeling

We investigate the volatility dynamics of the CBIOS contract traded at the Brazilian commodity exchange B3 in relation to the primary underlying future contracts and local spot prices through the use of Engle’s dynamic conditional correlation (DCC) (2002) methodology. This approach allows us to analyze the relationship between the various market variables and examine how their correlations change over time. This method gives insights into the complex interactions between the CBIOS contract, underlying future contracts, and spot prices and how they contribute to the market’s overall volatility.^1^

Engle (2002) developed a time-varying correlation with a multivariate generalized autoregressive conditional heteroskedasticity (GARCH) model with univariate GARCH flexibility coupled with parametric models for the correlations. The standard GARCH is defined as follows:

1 $${r}_{t}=\mu +\alpha {r}_{t-1}+{\varepsilon }_{{\text{t}}}$$

with standard residuals being given by ${\varepsilon }_{t}=\sqrt{{H}_{t}{z}_{t}}$. The model is a generalization of the constant conditional correlation (Bollerslev 1990), i.e.,

$${H}_{t}={D}_{t}R{D}_{t},$$

2 $${E}_{t-1}\left({\varepsilon }_{t}{\varepsilon }_{t}^{\prime}\right)={D}_{t}^{-1}{H}_{t}{D}_{t}^{-1}=R, {\text{ since }} {\varepsilon }_{t}={D}_{t}^{-1}{r}_{t}.$$

where ${H}_{t}$ is a conditional covariance matrix (*n* × *n*), *R* is the conditional correlation matrix, and ${D}_{t}$ is a diagonal matrix with time-varying standard deviations on the diagonal, allowing *r* to be time varying. The volatility univariate model is applied to estimate the correlation parameter

3 $${Q}_{t}=\left(1-a-b\right)\overline{P }+a{\varepsilon }_{t-1}{\varepsilon }_{t-1}^{\prime}+b{Q}_{t-1},$$

4 $${P}_{t}={Q}_{t}^{*-1}{Q}_{t}{Q}_{t}^{*-1}$$

where $\overline{P }=E[{\varepsilon }_{t}{\varepsilon }_{t}^{\prime}]$ and *a* and *b* are non-negative scalars. The DCC is mean reverting since *a* + *b* < 1. The diagonal matrix with the square root of the $i$ th diagonal element of ${Q}_{t}$ in its *i*th diagonal position is represented by ${Q}_{t}^{\ast}=\left[{q}_{iit}^{\ast}\right]={\left\lfloor{q}_{iit}\right\rfloor}$. Thus, the correlation estimator is a positive definitive matrix, ${Q}_{t}$, with a ${q}_{ijt}$ as a weighted average of a positive semidefinite matrix, given by

5 $${\uprho }_{{\text{ijt}}}=\frac{{q}_{ijt}}{\sqrt{{q}_{iit}{q}_{jjt}}}$$

### Optimal hedge ratio (HR)

Our study aims to evaluate the effectiveness of the CBIOS contract in hedging energy prices in Brazil. To achieve this, we applied the concept of hedge ratio (HR), which provides valuable insight into the number of contracts required to be sold to hedge an investor’s long position. Through this approach, we were able to assess the efficacy of the CBIOS contract in managing risk in the energy market. Moreover, we analyzed the returns of a hedged portfolio and investigated the relationship between the CBIOS contract and the variables involved. Specifically, we used the following equations to estimate the relationship:

6 $$\Delta {CBios}_{t}={\text{ln}}\left(\Delta {CBios}_{t}\right)-{\text{ln}}\left(\Delta CBios\right), \Delta {F}_{t}={\text{ln}}\left(\Delta {F}_{t}\right)-{\text{ln}}(\Delta {F}_{t}),$$

where ${F}_{t}$ is the underlying energy contract to hedge the CBIOS.

The HR can be obtained by minimizing^2^ the variance of $\Delta CBio$ with respect to H,

7 $${H}_{mv}=\frac{Cov(\Delta {CBios}_{t},\Delta {F}_{t})}{Var(\Delta {F}_{t})}$$

To construct the HR, we employ the conditional volatility estimated from the multivariate GARCH models (Kroner and Sultan 1993). Moreover, we apply the hedge effectiveness (HE) obtained from a GARCH model to measure the optimal HR performance—higher HE suggests a better hedging portfolio (Basher and Sadorsky 2016). To build the hedge portfolio, we forecast the HR using a rolling window of 200 one-step ahead (1 day ahead).

### Data

We collected daily spot prices for CBIOS from the Brazilian exchange market (B3), sugar, and ethanol prices from CEPEA (Centre for Advanced Studies on Applied Economics), Brazil, and Brent from the Energy Information Agency (EIA), USA. Additionally, we collected future contract prices for Brent oil, ethanol Platts Chicago, and sugar NY from Bloomberg. The dataset covers the period from August 3, 2020, to June 30, 2022, with 456 observations. We transformed the dataset into the log-return form: ${r}_{i}={\text{ln}}{p}_{t}-{\text{ln}}{p}_{t-1}$.

## Empirical results

**Fig. 1:**
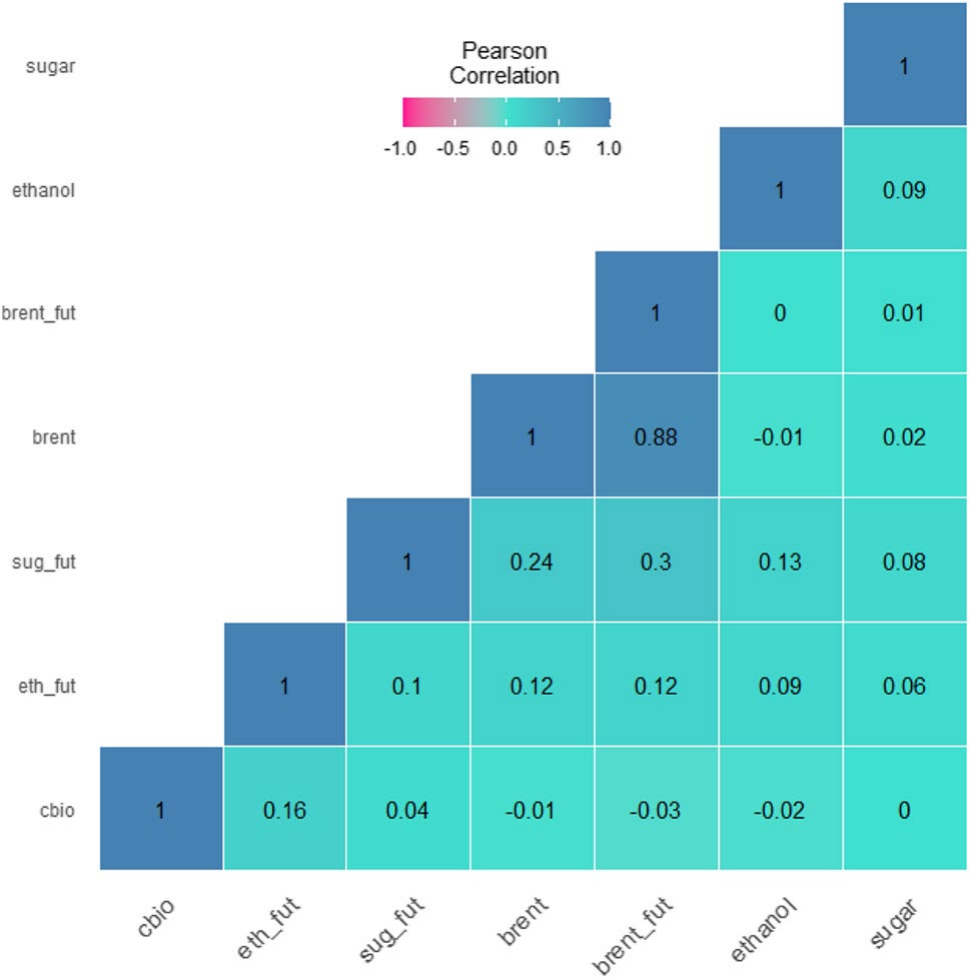
Pearson correlation matrix of cash price log return of Brent, CBIOS, ethanol, and sugar and future contracts of Brent, sugar (NY), and ethanol Chicago (Platts) from August, 2020, to June, 2022

**Fig. 2:**
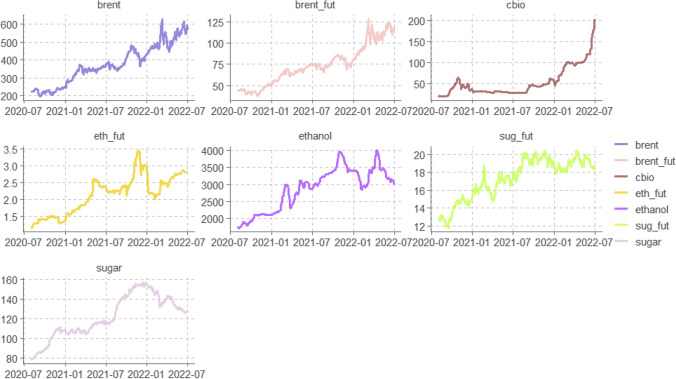
Time series plots of the involved variables in levels from August, 2020, to June, 2022

Table 1 displays the descriptive statistics of the time series. The CBIOS and Brent prices exhibit the highest standard deviation. The presence of ARCH effects across all variables is confirmed by rejecting the null hypothesis in the ARCH-LM test. Figure 1 presents the time series plots of the variables, while Fig. 2 shows the Pearson correlation in log returns. The analysis reveals a low correlation between ethanol and sugar spot prices, which reflects the distinctive characteristics of the sugarcane industry in Brazil. Sugar mills have the flexibility to produce either sugar or ethanol, depending on market conditions, with a typical ratio of around 60% to 40% (Palazzi et al. 2022).

**Table 1:** Daily return statistic summary

	CBIOS	brent	ethanol	sugar	sug_fut	brent_fut	eth_fut
Min	− 0.26	− 0.14	− 0.07	− 0.06	− 0.08	− 0.14	− 0.32
Max	0.16	0.19	0.04	0.03	0.06	0.14	0.14
Range	0.42	0.33	0.11	0.09	0.14	0.28	0.46
Sum	2.27	0.96	0.53	0.48	0.39	0.96	0.87
Median	0.00	0.00	0.00	0.00	0.00	0.00	0.00
Mean	0.00	0.00	0.00	0.00	0.00	0.00	0.00
Jarque–Bera	^***^	^***^	^***^	^***^	^***^	^***^	^***^
ARCH test	^***^	^***^	^***^	^*^	-	^***^	-
Std. dev	0.03	0.03	0.01	0.01	0.02	0.03	0.03
Coef. var	6.79	13.07	9.70	9.19	19.54	12.13	13.28
Skewness	− 1.64	0.06	− 0.22	− 0.75	0.01	− 0.28	− 4.99
Kurtosis	20.98	9.62	7.35	6.49	3.97	8.28	67.94

Source: author compilation

**Fig. 3:**
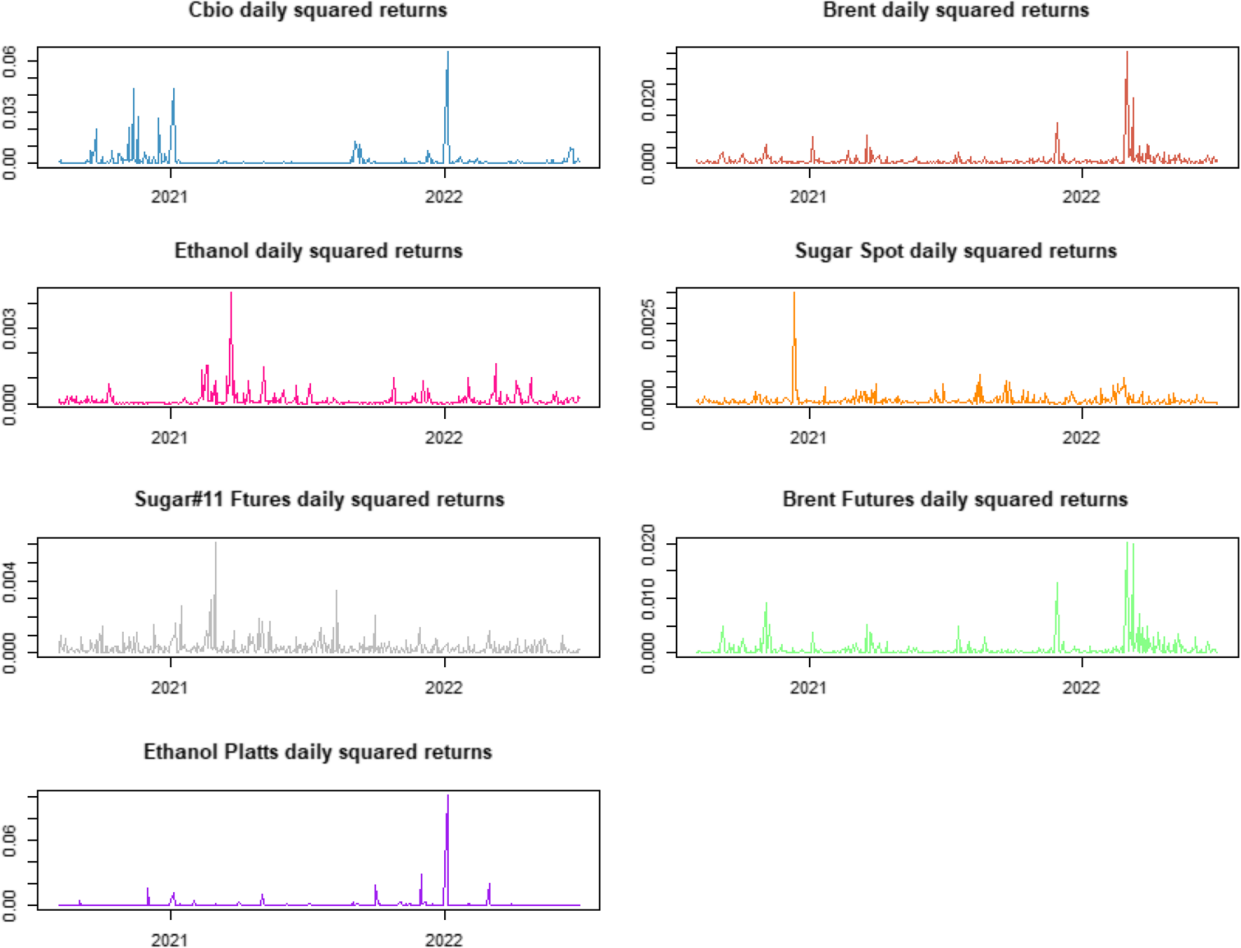
Squared daily returns of Brent, CBIOS, ethanol, and sugar spot and future prices

We further provide the squared returns time series graphs depicted in Fig. 3, which demonstrate the dynamic patterns of volatility over time, revealing periods of volatility clustering.

**Fig. 4:**
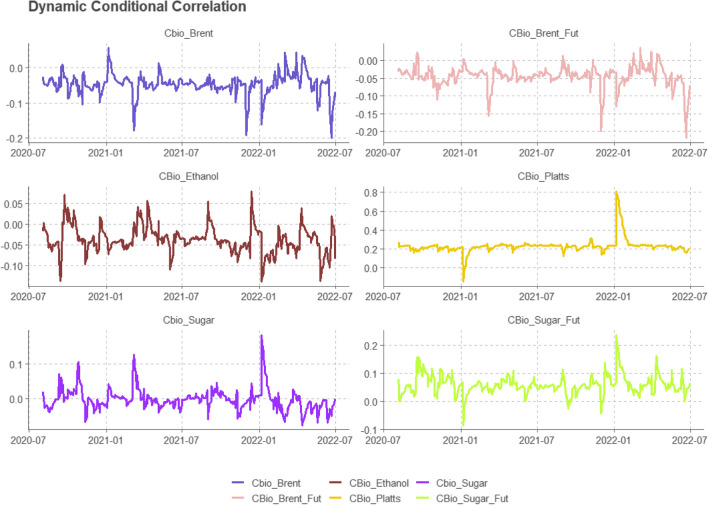
The dynamic conditional correlation using the DCC-GARCH model

The first step in building the DCC-GARCH model was to select the best distribution of a GARCH(1,1) variance equation with a constant in the mean equation. We selected a multivariate normal distribution according to the best information criteria (BIC and AIC). Further, we estimated a 200 one-step-ahead DCC using a rolling window, and the GARCH model is refitted every 10 observations. Figure 4 shows the dynamic correlation between CBIOS and the other variables.

The pairwise dynamic correlation between CBIOS and ethanol Platts is positive for the most part, reaching the highest peak of 81% in June, 2022. On the other hand, around January, 2021, the correlation reached its lowest value (− 15%). The correlation between the sugar future price and CBIOS is also positive, with a lower correlation value than CBIOS-Platts. For both ethanol Platts and sugar future contracts, the highest correlation happened in early 2022, reaching 81% and 24%, respectively. Spot and future Brent prices presented a negative correlation with CBIOS for most of the sample. In contrast, the correlation between ethanol and sugar cash prices with CBIOS remained steady at around zero.

**Fig. 5:**
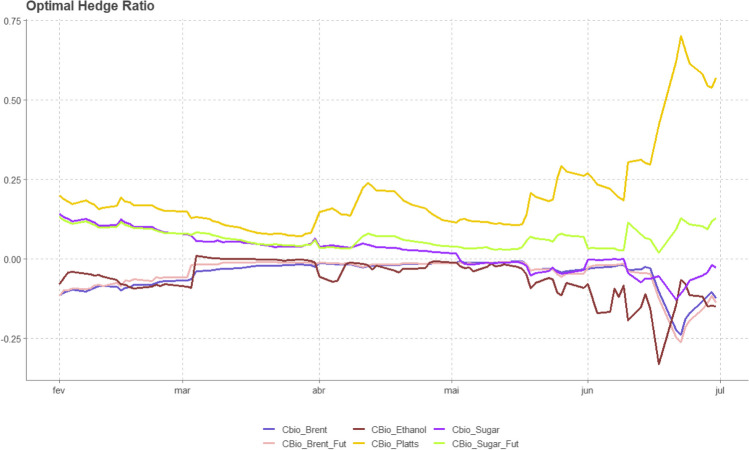
Optimal hedge ratio using a rolling window of 200 one-step forecasts

Figure 5 illustrates the time-varying optimal hedge for CBIOS. The results indicate that, apart from the sugar and ethanol Platts future prices, the remaining contracts provide a lower opportunity for hedging CBIOS. Table 2 confirms the low hedge effectiveness of these contracts in serving as a hedging instrument for CBIOS. However, the Platts and sugar futures exhibit an increasing trend, indicating a potentially viable candidate for hedging opportunities despite the limited existence of CBIOS.

**Table 2:** Hedge ratio summary and hedging effectiveness (HE)

	Mean	Min	Max	HE
CBIOS-Brent	− 0.05	− 0.24	− 0.01	− 0.02
CBIOS-ethanol	− 0.06	− 0.33	0.01	− 0.01
CBIOS-sugar	0.02	− 0.13	0.14	− 0.01
CBIOS-sugar futures	0.07	0.02	0.13	− 0.01
CBIOS-Brent futures	− 0.04	− 0.26	− 0.01	0.02
CBIOS-ethanol Platts	0.20	0.07	0.70	0.00

Notably, ethanol results were divergent. It is to be expected that, due to the sample limitation, variations in correlations will arise between CBIO and Platts ethanol and CBIO and Brazilian ethanol. In the short term, evidence suggests divergence in Brazilian and American ethanol prices, while in the long term, a trend of price convergence becomes apparent (Dutta 2020; Hernandez et al. 2020; Quintino et al. 2021b). Despite the positive correlation between Brazilian ethanol and international oil prices, a large part of the Brazilian ethanol supply is absorbed by domestic market demand, which tends to limit the transmission of international price shocks to domestic prices in the short term. The lower correlation between CBIOS prices and ethanol prices is in line with the results of Quintino et al. (2021a), who studied the EU Allowance (EUA) emission prices and Brazilian ethanol prices and showed that the relationship is weak and short term.

Regarding the sugar prices, the results were very similar. It is expected that their prices will have stronger convergence with each other, including in the short term, given that Brazil is the largest global supplier and, therefore, its supply has a significant impact on international prices (Amrouk and Heckelei 2020). Rumánková et al. (2019) found evidence that the main international sugar prices, such as New York and London, despite their regional differences and technical specificities, have a significant interrelationship, with Brazil and India as key players in this interdependence due to their supply capabilities.

Unlike the main EU Allowance (EUA) emissions market, which exhibits a strong and growing positive correlation with Brent oil prices—as documented by Lee and Yoon 2020)—our analysis reveals no significant association between CBIO prices and Brent oil. This divergence suggests that CBIOS prices may be less sensitive to external factors, potentially due to the market’s nascent stage. Characterized by limited liquidity and concentrated ownership within the fuel distribution sector, the CBIOS market may not yet exhibit the price dynamics observed in more mature carbon markets.

## Conclusions and policy implications

The CBIO program was designed to incentivize biofuel production and promote environmental sustainability in line with the transition to a low-carbon economy. This study examines the viability of the contract in financial markets, as its success may depend on the adherence of an underlying asset in commodity markets. The availability of a liquid and efficient market for trading CBIO contracts can increase the attractiveness of CBIOs as an investment and risk management tool for market participants.

Our results indicate a low level of hedge effectiveness between CBIO and sugar, Brent, and Brazilian ethanol prices. However, the dynamic optimal hedge analysis reveals an increasing relationship between CBIO and ethanol Platts over time, suggesting a potential instrument for hedging. These findings are important not only in identifying a potential instrument for hedging and investors’ interest in the transition toward a cleaner economy but also in emphasizing the need for investors and policymakers to manage risks associated with energy prices and decarbonization efforts effectively.

Furthermore, this study could be relevant to other countries, as it highlights the importance of policies that promote sustainable energy and their potential impact on financial markets. Such policies can contribute to a more sustainable environment by promoting the use of green energy in substitution for fossil fuel energy.

Thus, our study has several policy implications worth noticing. The knowledge of hedging effectiveness helps attract a larger number of participants interested in operating in this market, which would enhance liquidity and improve market efficiency with greater fluidity of information among stakeholders. The fuel distribution market in Brazil has hundreds of companies; however, considering the year 2022, the three largest companies (Vibra, Raízen, and Ipiranga) held approximately 63.7% of the market and, consequently, the obligation to purchase CBIOS (NovaCana 2022b).

Hence, the success of the CBIOs program has the potential to foster a more competitive environment for biofuel prices, particularly in competition with gasoline prices. This success could be attributed to incentives encouraging the expansion of biofuel supply. As concerns arise about the adequacy of the national ethanol supply derived solely from sugarcane, there is a possibility of supplementing the supply with corn ethanol. This strategic move aims to discourage the substitution of ethanol with gasoline, promoting the sustainability and viability of the biofuel market (Gonçalves et al. 2023).

Additionally, the CBIO program could encourage the provision of more accurate information to the ethanol consumer, as suggested by Nascimento Filho et al. (2022), which is a federal law that determines the establishment of information on the ethanol-gasoline price ratio at gas stations, as well as encouraging knowledge of the energy efficiency of your own vehicle. The ethanol-gasoline relative price ratio, in certain situations, particularly when the relative price is close to 0.7, is very sensitive to the choice of the best alternative. For example, a small change in the price ratio in favor of ethanol could lead to large positive changes in the amount of biofuel demand (Nascimento Filho et al. 2022).

Future studies could benefit from an extended time frame as the CBIOS market matures. This would allow for a more comprehensive analysis of price interdependence under diverse national and international macroeconomic conditions. As evidenced by Uddin et al. (2021), geopolitical risks, macroeconomic uncertainties, financial stress, and market volatility significantly influence biofuel prices, including those of Brazilian ethanol. Therefore, an extended timeline would enable researchers to capture a wider range of these interdependencies and potentially refine our understanding of how they shape CBIOS market dynamics.

## Figures and Tables

**Fig. 6:**
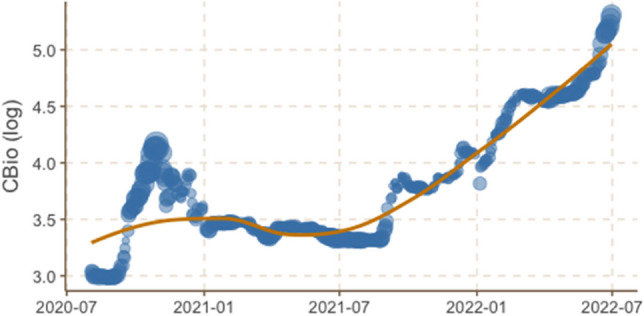
Scatter plot of the CBIO (dependent variable), the future contracts (Brent, sugar, and ethanol), and the spot variables (sugar and Brent)

## Data Availability

Daily spot prices for CBIOS from the Brazilian exchange market (B3), sugar and ethanol prices from CEPEA (Centre for Advanced Studies on Applied Economics), Brazil, and Brent from the Energy Information Agency (EIA), USA. Future contract prices for Brent oil, ethanol Platts Chicago, and sugar NY from Bloomberg.

## Appendix

### A. Model specification

To support our conclusion and the methodology approach, we define in this section the relation between the variables involved. We start by regressing the log fuel price variables as independent variables about the log dependent variable (CBIOS) as follows:

$$CBio=\alpha + {\beta }_{1}Bren{t}_{spot}+ {\beta }_{2}Suga{r}_{spot}+{\beta }_{3}Suga{r}_{future}+{\beta }_{4}Bren{t}_{future}+{\beta }_{5}Ethano{l}_{future}+\epsilon$$

**Table 3:** Regression analysis

	*Dependent variable:*
	log(CBIO)
log(Brent)	-0.822** (0.373)
log(Ethanol)	-2.210*** (0.274)
log(Sugar)	2.280*** (0.226)
log(S*ugarfutures)*	-0.805** (0.382)
*log(Brentfutures)*	2.487*** (0.333)
log(*Ethanolf utures)*	0.202 (0.154)
Constant	6.828*** (1.501)
Observations	456
R2	0.639
AdjustedR^2^	0.634
Residual Std. Error	0.324 (df = 449)
F Statistic	132.415*** (df = 6; 449)

**p* < 0. l; ***p* < 0.05; ****p* < 0.01

Appendix Fig. 6 depicts the relationship between CBIO and involved variables in the model through a linear regression model. The regression line, shown in orange, represents the best-fit line that minimizes the sum of squared differences between observed data points and predicted values. Appendix Table 3, in turn, shows the results of the multiple regression. Notably, all the variables are significant except for the ethanol futures.

In addition, we include the tests for heteroskedasticity in the conditional variance and the parameters estimated from the DCC-GARCH model. For instance, Appendix Table 4 illustrates the ARCH test to evaluate whether the volatility of the series is constant over time. We find evidence that for all variables, except sugar spot and futures and ethanol futures, the presence of conditional heteroskedasticity. The results indicate the use of the GARCH approach to deal with the conditional variance inconsistency over time.

**Table 4:** ARCH test (autoregressive conditional heteroskedasticity)

	Test statistic	*p*-value	Result H0
CBIO	38.33	0.00	Reject H0
Brent	56.02	0.00	Reject H0
Ethanol	79.23	0.00	Reject H0
Sugar	19.80	0.07	Fail to Reject H0
Sugar futures	15.06	0.24	Fail to Reject H0
Brent futures	64.11	0.00	Reject H0
Ethanol futures	0.33	1.00	Fail to Reject H0

The test determines whether the null hypothesis of constant variance is rejected for each variable, indicating the presence of heteroskedasticity

Moving to Appendix Table 5, we analyze the optimal parameters in the model. $\alpha$ and $\beta$ show how volatility evolves over time, with $\alpha$ capturing the short-term volatility and $\beta$ the long-term volatility. The long-term parameter is higher than the alpha for the commodities traded in the future markets, which illustrates the commodity time series behavior.

**Table 5:** Optimal parameters DCC-GARCH fitting model

Optimal parameters	Estimate	Std. error	*t*-value	Pr( >|*t*|)
CBIO $\mu$	0.00	0.01	0.02	0.99
CBIO $\omega$	0.00	0.00	0.51	0.61
CBIO $\alpha$	0.18	0.17	1.10	0.27
CBIO $\beta$	0.82	0.06	14.16	0.00
Brent $\mu$	0.00	0.00	2.56	0.01
Brent $\omega$	0.00	0.00	2.70	0.01
Brent $\alpha$	0.10	0.04	2.30	0.02
Brent $\beta$	0.80	0.05	15.17	0.00
Ethanol $\mu$	0.00	0.00	1.58	0.11
Ethanol $\omega$	0.00	0.00	2.86	0.00
Ethanol $\alpha$	0.73	0.17	4.24	0.00
Ethanol $\beta$	0.26	0.08	3.07	0.00
Sugar $\mu$	0.00	0.00	2.00	0.05
Sugar $\omega$	0.00	0.00	2.01	0.04
Sugar $\alpha$	0.11	0.04	2.69	0.01
Sugar $\beta$	0.72	0.12	5.90	0.00
Sugar_fut $\mu$	0.00	0.00	0.81	0.42
Sugar_fut $\omega$	0.00	0.00	0.01	0.99
Sugar_fut $\alpha$	0.02	0.09	0.17	0.86
Sugar_fut $\beta$	0.98	0.10	10.30	0.00
Brent_fut.$\mu$	0.00	0.00	2.61	0.01
Brent_fut $\omega$	0.00	0.00	2.57	0.01
Brent_fut $\alpha$	0.15	0.05	2.98	0.00
Brent_fut $\beta$	0.78	0.04	19.74	0.00
Ethanol_fut $\mu$	0.00	0.00	1.11	0.27
Ethanol_fut $\omega$	0.00	0.00	0.99	0.32
Ethanol_fut $\alpha$	0.03	0.03	1.21	0.23
Ethanol_fut $\beta$	0.95	0.02	45.61	0.00
Jointdcca1	0.02	0.01	1.93	0.05
Jointdccb1	0.80	0.06	13.26	0.00

Joint parameters Jointdcca1 and Jointdccb1 represent the DCC coefficients for the entire system, where *μ* represents the mean, *ω* is the volatility intercept, *α* is the ARCH coefficient, and *β* is the GARCH coefficient

## Footnotes

1 See Appendix for more information about the estimation and the relationship between the variables.

2 For further references on optimal HR, see Baillie and Myers (1991)
